# Bilateral Pneumonia in a Patient with Chronic Bronchiectasis Caused by Achromobacter xylosoxidans Subspecies denitrificans

**DOI:** 10.7759/cureus.7381

**Published:** 2020-03-23

**Authors:** Gauthier Stepman, Kulveer Dabb, Imran A Khan, Jordan T Young, Johnathan Frunzi

**Affiliations:** 1 Internal Medicine, Medical Center of Trinity, Trinity, USA; 2 Pulmonology, Medical Center of Trinity, Trinity, USA

**Keywords:** achromobacter, xylosoxidans, denitrificans, bronchiectasis, pneumonia

## Abstract

*Achromobacter xylosoxidans* is a gram-negative bacillus that has a multitude of inherent and acquired antimicrobial resistance. It is a rare, isolated pathogen in patients without cystic fibrosis (CF). We report the case of a 76-year-old Caucasian male with a history of chronic obstructive pulmonary disease (COPD), previous* Mycobacterium-avium intracellulare* (MAI) infection, and chronic bronchiectasis who did not respond to three courses of outpatient antibiotics for a chronic cough. He also had a 21-lb weight loss. The diagnosis of *Achromobacter xylosoxidans* subspecies *denitrificans* was made through bronchoscopy with bronchoalveolar lavage (BAL).

There are few case reports describing *Achromobacter xylosoxidans* subspecies *denitrificans* in non-CF patients. *Achromobacter xylosoxidans *colonization might be linked to predisposing lung damage such as in CF and bronchiectasis. The bacterium is frequently multidrug-resistant. More studies are needed to develop recommendations for clinical guidelines to address the increasing antibiotic resistance to *Achromobacter xylosoxidans.*

## Introduction

Achromobacteria are gram-negative, motile bacilli that are oxidase-positive, catalase-positive, and lactose nonfermenting [1-2]. They are often found in aquatic environments and soil [1]. The bacterium was first described by Yabuuchi and Ohyama in 1971, when it was isolated from otic discharge [3]. Several subspecies have been identified since its discovery in 1971, of which most are nonpathogenic [1-2]. Some subspecies, however, can cause significant disease in patients with predisposing tissue damage such as in cystic fibrosis (CF) or bronchiectasis. The most clinically significant subspecies are *xylosoxidans *and *denitrificans *[2,4]. It is thought that bacteremia with this organism is most likely to occur nosocomially and more specifically in immunocompromised patients [5]. One study found in a 10-year follow-up that solid organ cancers and heart failure were most commonly associated with *Achromobacteria (A.) xylosoxidans* bacteremia [6]. Pulmonary infections, however, are independent of a patient’s immunocompetency but are believed to be related to lung tissue integrity [5,7].

*A. xylosoxidans* subspecies *denitrificans *is less commonly reported as a pathogen than the *xylosoxidans *subspecies [2,4]. *A. xylosoxidans *is a relatively rare pathogen to cause respiratory disease but is more commonly found in patients with CF [8]. The reported prevalence rate of colonization in CF populations varies between 5.3% and 13.1% [8-9]. This case report describes a patient with a past medical history of chronic bronchiectasis who was diagnosed with pneumonia caused by *A. xylosoxidans* subspecies *denitrificans*.

## Case presentation

**Figure 1:**
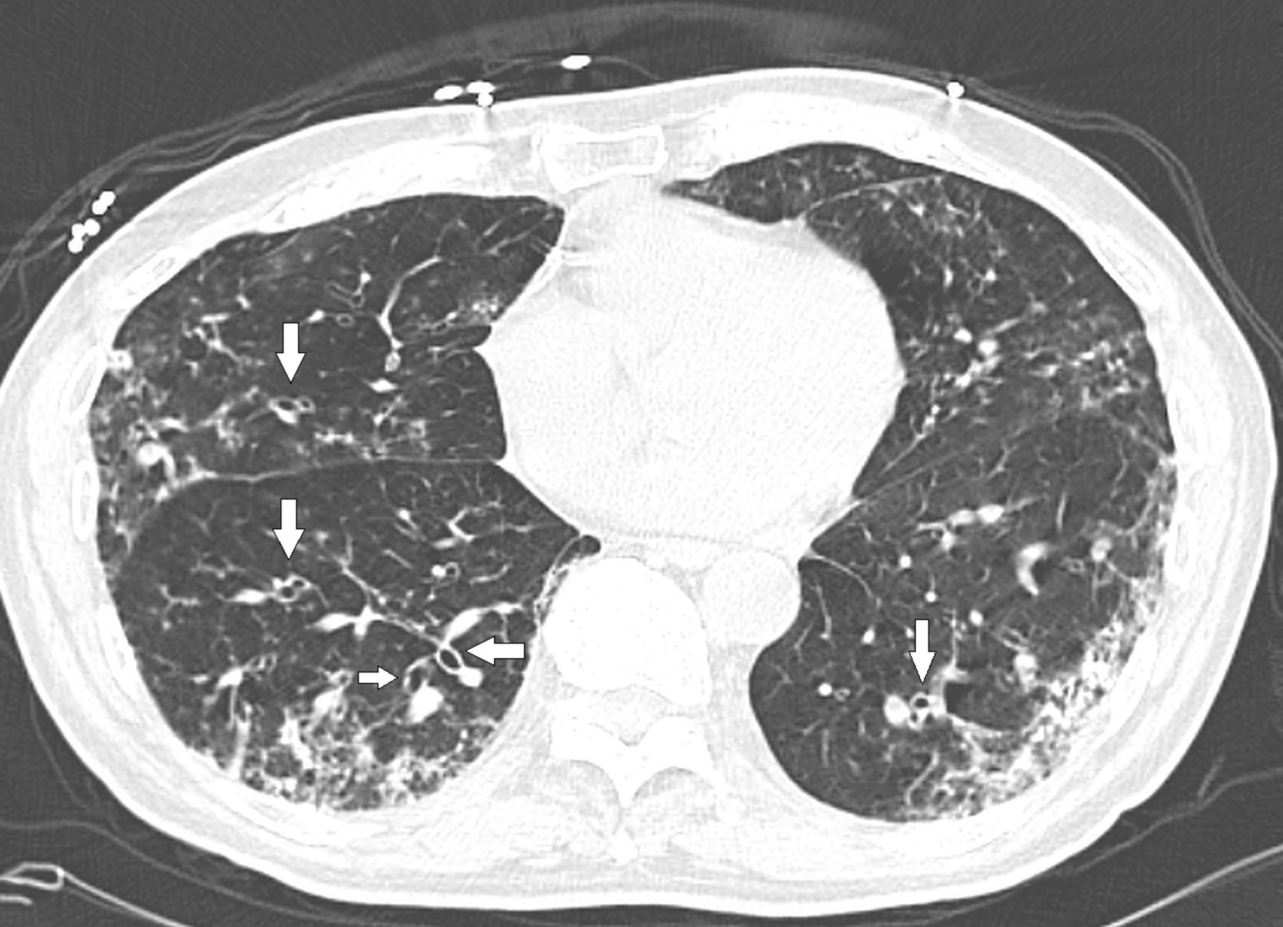
Computed tomography scan showing bronchiectasis

**Figure 2:**
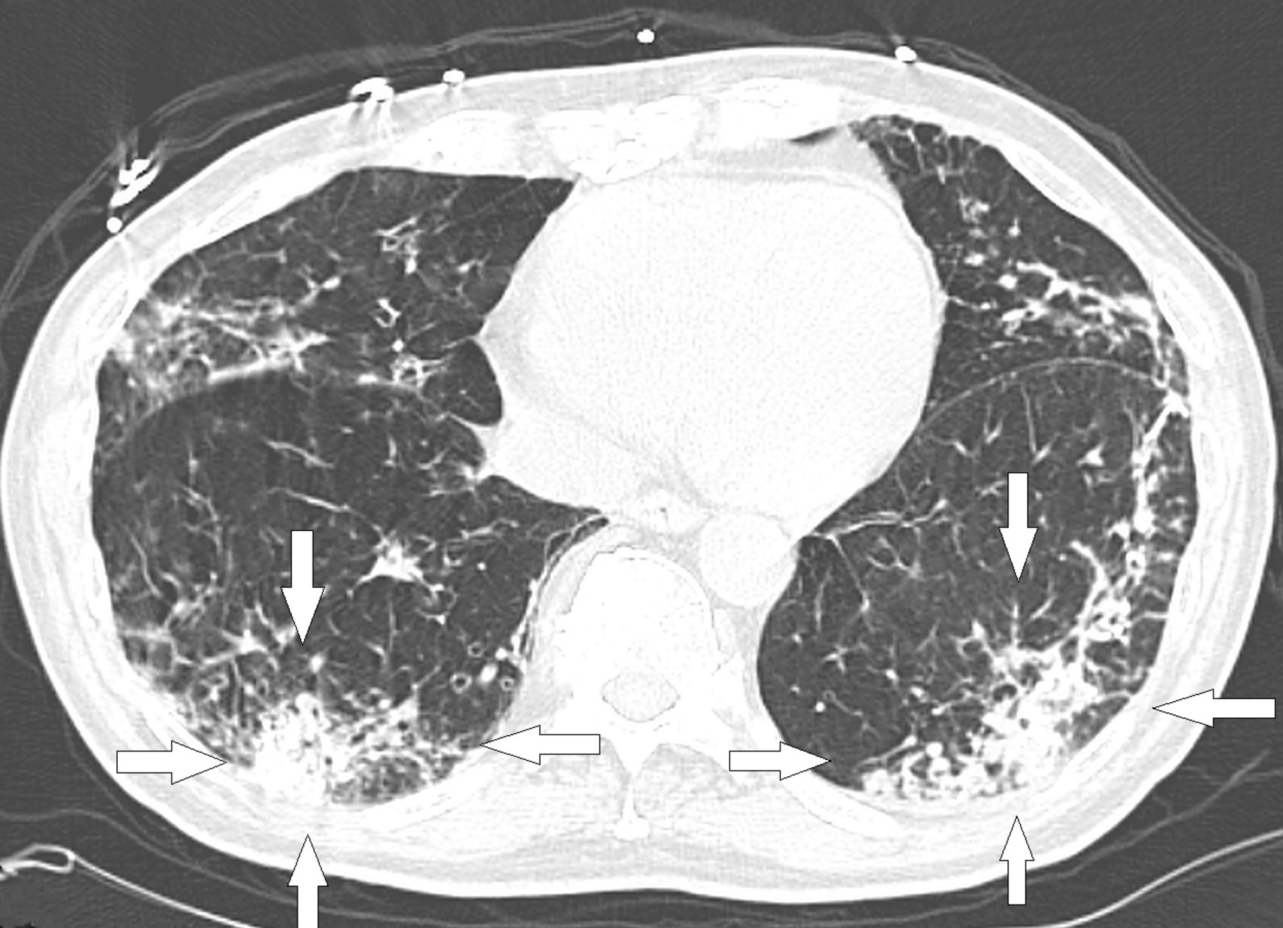
Computed tomography scan showing bilateral lower lobe consolidation

Our patient is a 76-year-old Caucasian male with a past medical history significant for chronic obstructive pulmonary disease (COPD), pulmonary *Mycobacterium avium-intracellulare* (MAI) infection, and bronchiectasis. He presented to his pulmonologist’s office with complaints of persisted productive cough, subjective fevers, rhinitis, and a 21-lb unintentional weight loss over a period of three months. The patient was started on a seven-day course of doxycycline. However, doxycycline did not improve his cough. He was then prescribed a second course of doxycycline by his primary care physician, again without any clinical improvement. After completion of the second course of antibiotics, he was prescribed cefdinir 300 mg, twice daily for seven days. Due to the continued symptoms after his third antibiotic course, he was admitted to the hospital for further evaluation. During his hospitalization, a computed tomography (CT) scan was performed, which showed evidence of bronchiectasis (Figure 1) and bilateral lower lobe consolidations (Figure 2).

He was started on intravenous (IV) ceftriaxone 1 gram daily and oral azithromycin 500 mg daily. Given his history of MAI infection and current prolonged history of coughing despite multiple courses of antibiotics, the patient was evaluated for immunodeficiency. Laboratory tests for immunoglobulins revealed normal to above-normal levels (see Table 1).

**Table 1:** Immunoglobulin levels in our patient

Immunoglobulin	Level (mg/dL)	Reference Range (mg/dL)
IgG	1997	700 – 1600
IgG1	1072	248 – 810
IgG2	500	130 – 555
IgG3	104	15 – 102
IgG4	105	2 – 96
IgA	461.9	70 – 400
IgM	48	40 – 230

Culture of the bronchoalveolar lavage revealed the presence of *A. xylosoxidans* subspecies *denitrificans *in both lower lobes. The lavage was also sent for acid-fast bacilli, which confirmed a recurrence of MAI. Antibiotic sensitivity tests for the *A. xylosoxidans* subspecies *denitrificans *strain in this patient is documented in Table 2.

**Table 2:** Microbiological sensitivities of isolated Achromobacter species

Antimicrobial agent	Presence of sensitivity	MIC
Gentamicin	Intermediate	8
Tobramycin	Sensitive	4
Ciprofloxacin	Resistant	>=4
Ceftazidime	Intermediate	16
Amikacin	Sensitive	16
Cefepime	Resistant	>=64
Piperacillin/Tazobactam	Sensitive	<=4
Meropenem	Sensitive	1

The patient’s antibiotic regimen was switched to IV meropenem due to the sensitivity test results. A peripherally inserted central catheter (PICC) was inserted and the patient was discharged with a two-week course of IV meropenem. The patient was seen two weeks after discharge by his primary care physician and five weeks after discharge by his pulmonologist. He reported feeling better with his cough being improved. He also reported some weight gain since his last visit.

## Discussion

*A. xylosoxidans* is a rare cause of pneumonia in the immunocompetent adult. Infections with *A. xylosoxidans*, subspecies *denitrificans *are less common than with the *xylosoxidans *subspecies. Swenson and Sadikot reviewed 32 case reports of respiratory infections with* A. xylosoxidans* in patients without CF and found only three cases in which *A. xylosoxidans*, subspecies *denitrificans *was the causative organism [4]. We reviewed all current reported cases of pneumonia caused by* A. xylosoxidans* subspecies *denitrificans *and reported this in Table 3.

**Table 3:** Current case reports with Achromobacter xylosoxidans subspecies denitrificans as causative organism IV: intravenous; COPD: chronic obstructive pulmonary disease; MAI: Mycobacterium avium-intracellulare

Year reported	Age, sex	Clinical syndrome	Isolation specimen	Comorbid conditions	Sensitivity	Antibiotic of choice	Treatment duration
2012 [10]	Not reported	Pneumonia	Tracheal aspirate	Colon cancer	Ceftazidime, piperacillin/tazobactam, cefoperazone-sulbactam, levofloxacin, imipenem, meropenem, tigecycline	Piperacillin/tazobactam	14 days
2012 [11]	50, female	Pneumonia	Bronchoalveolar lavage	Adrenal insufficiency, caused by pneumonia	Ceftazidime, ciprofloxacin, ofloxacin, piperacillin/tazobactam, cefoperazone/sulbactam	IV ciprofloxacin	14 days
2014 [12]	55, male	Bronchopneumonia	Bronchoalveolar lavage	Bronchiectasis	Not reported	Not reported	Not reported
2014 [5]	48, male	Pneumonia	Sputum	History of tuberculosis	Meropenem, imipenem, piperacillin, ticarcillin, trimethoprim-sulfamethoxazole, third-generation cephalosporins	Meropenem 1g q 8 hours	14 days
2017 [2]	45, female	Pneumonia	Bronchoalveolar lavage	Bronchiectasis, asthma, gastroesophageal reflux disease	Amikacin, cefepime, ceftazidime, gentamicin, levofloxacin, meropenem, piperacillin/tazobactam, tobramycin, trimethoprim/sulfamethoxazole	Levofloxacin	6 weeks
2019 [13]	69, male	Pneumonia	Sputum	COPD, bronchiectasis	Piperacillin/tazobactam, ceftazidime	Piperacillin/tazobactam	14 days
2020	76, male	Current case; Pneumonia	Bronchoalveolar lavage	MAI-colonization, bronchiectasis	Tobramycin, amikacin, piperacillin/tazobactam, meropenem	Meropenem 500 mg q 8 hours	14 days

The presentation of *A. xylosoxidans* pneumonia is atypical. Common symptoms are fever, productive cough, weight loss, progressive shortness of breath, and fatigue [2,5,14]. The prolonged clinical course and resistance to multiple antibiotic treatments warrant further investigations.

Since *A. xylosoxidans* is most frequently associated with CF, it is believed that pre-existing lung damage makes patients more susceptible to colonization or infection with *A. xylosoxidans* [4,6,8]. Our patient had chronic bronchiectasis, most likely due to a previous MAI infection. This was similar to the patient described by Bharadiya et al., who had bronchiectasis secondary to previous mycobacterial infection [5]. In fact, most of the case reports that describe *Achromobacter xylosoxidans*, subspecies *denitrificans *pneumonia were in patients with bronchiectasis [2,13-14].

Microscopically, *A. xylosoxidans* is very similar to *Pseudomonas aeruginosa*, which can potentially lead to lower rates of detection [8]. In patients with CF, the co-colonization of *P. aeruginosa *and* A. xylosoxidans* was associated with a significant decline in respiratory function [9]. Since patients with pre-existing lung damage are often colonized with *P. aeruginosa*, it was suggested to systematically eradicate *A. xylosoxidans*, as is currently recommended for *P. aeruginosa* [9,14]. This could potentially prevent a decline in respiratory function in patients with non-CF *A. xylosoxidans* infections.

*A. xylosoxidans *is frequently a multidrug-resistant organism (MDRO). The multidrug resistance is broad, however, and not uniform between different isolates, making antibiotic choice challenging [4].

A 10-year case series of *A. xylosoxidans* bacteremia noted that most isolates were susceptible to meropenem and piperacillin or tazobactam [7]. This was also the case for our patient. Current guidelines for the treatment of *A. xylosoxidans* infections in patients with bronchiectasis are not available. The current treatment practices are mainly based on bronchiectasis and *P. aeruginosa* treatment [13].

## Conclusions

The purpose of this case report is to inform physicians that patients with pneumonia, resistant to multiple antibiotics, need further investigations to identify the causative organism. This is especially true for patients with predisposing lung tissue damage, as in bronchiectasis and CF. Due to the microbiological similarities, treatment practices currently rely on *P. aeruginosa* infections. More studies are needed to form guidelines on management. Since *A. xylosoxidans* is an uncommon MDRO pathogen to cause disease in non-CF patients, patients often fail multiple outpatient antibiotic treatments. The need for intravenous antibiotics is high, making outpatient treatment difficult. The MDRO nature of *A. xylosoxidans* makes it important to timely identify the pathogen as to not further increase resistance, especially in patients with lung damage, who are at increased risk. More studies are also needed to evaluate the influence of *Achromobacter xylosoxidans* respiratory infections on the pulmonary function.
